# Peer Review of “The Psychological Impact of Hypertension During COVID-19 Restrictions: Retrospective Case-Control Study”

**DOI:** 10.2196/28714

**Published:** 2021-03-30

**Authors:** Jorge Andrés Delgado-Ron

**Affiliations:** 1 Health and Community Design Lab School of Population and Public Health The University of British Columbia Vancouver, BC Canada

**Keywords:** public health, global health, COVID-19, hypertension, risk, strategy, mental health, behavior, response, anxiety, vaccine, retrospective, perception, prevention, intention

*This is a peer review submitted for the paper “The Psychological Impact of Hypertension During COVID-19 Restrictions: Retrospective Case-Control Study.”*

## Round 1 Review

### General Comments

This study [1] aimed to investigate whether Australians with hypertension have higher risk perceptions, anxiety, and prevention intentions than Australians without hypertension during COVID-19 restrictions in April and June 2020. The authors used a national survey subsample (those who reported hypertension and not other comorbidities). They matched them with controls using age, gender, education, and health literacy. This is a nationally representative sample that includes several dimensions of an individual’s mental health. The question is relevant for future public health interventions.

Overall, the study has several weaknesses and does not appropriately answer the study aim because the reported results are not consistent with the proposed methods. The authors also failed to address alternative explanations to their findings. Please see my detailed feedback after the minor comments.

### Specific Comments

#### Major Comments

1. There is a major disconnect between the proposed methods and the results. Moreover, the authors need to clarify the assumptions that led to the selection of their methods.

2. The overall organization can improve. Some methods are presented in the *Results* or *Discussion* section, and some discussion points are introduced in the *Results* section.

3. The authors need to rewrite the *Introduction* section to better contextualize the potential mediators between exposure and outcome with relevant literature.

4. The authors need to rewrite the discussion emphasizing their findings and addressing their limitations and alternative explanations to their study results.

#### Minor Comments

1. The tables need to be reworked to not confuse multiple regression and marginal mean difference (MMD).

2. Tables are stand-alone pieces. Some of the methodologies need to be incorporated as a footnote.

3. Some typos need to be fixed across the manuscript.

4. Ethics need to be clarified (not a main concern as this is a secondary analysis).

#### Detailed Feedback:

##### Title/Abstract and References

1. Ideally, the title needs to include the study design, the population (Australia), and the study’s specific outcomes. Please consider changing it to better reflect your primary exposure: hypertension (eg, “The Impact of Hypertension on Adults’ Anxiety During COVID-19 Restrictions”).

2. The paper has relatively few references (15); some are press articles (3). The authors could strengthen their writing by considering some of these references:

- https://doi.org/10.1093/eurpub/cky114
- https://doi.org/10.1586/14760584.2015.964212
- https://apps.who.int/iris/bitstream/handle/10665/ 251671/WHO-HIS-TTi-GAP-16.2-eng.pdf

##### Introduction

1. The introduction is just one paragraph long. It discusses why hypertensive people could experience increased levels of COVID-19–related anxiety. However, it misses critical points at the center of this debate during the pandemic’s early stages (time of the survey). For instance, the role of antihypertensive medication as a potential risk factor on those infected by SARS-CoV-2:

- https://pubmed.ncbi.nlm.nih.gov/32737124/
- https://doi.org/10.1056/NEJMoa2007621

and existing studies on risk perception among people with chronic disease:

https://journals.plos.org/plosone/article?id=10.1371/ journal.pone.0237296

2. The research question would be clear and justified if the points considered above are included. I suggest adding details about the population (country).

##### Methods

1. The data selection process is clear *after* one reads the whole paper but not after reading the *Methods* section. I suggest mentioning early on that subjects with additional comorbidities were excluded from the sample. There is no mention of the matching method used and whether this was done manually or automatically (“randomly matched” is mentioned, but what type of randomization was used?). I would also add a line about (a) why you selected these covariates and (b) the test used to assess an adequate balance between the matched pairs.

2. There is no mention of ethics approval for this study. I understand the original survey was approved by the University of Sydney Human Research Ethics Committee (2020/212). Please mention whether this study is covered under the same authorization.

3. There is no mention of the absence or presence of systematic differences between the followed-up sample and those who decided not to participate for a second time. Was this tested? If there are differences, what are the potential implications?

4. Exposure: Please mention the definition of exposure in the *Methods* (self-reported).

5. Covariates: The *Methods* section reports using the health literacy single-item screener and the Consumer Health Activation Index patient activation measure. I understand these are validated tools. Please add a line about what these tools measure and why they are relevant to the current analysis.

6. Outcomes: Please detail more about risk perceptions and prevention behaviors in the *Methods* section.

7. Statistical analysis: (a) The use of “linear models for continuous outcomes, generalized linear models with modified Poisson approach for dichotomous outcomes, [and] ordinal logistic regression for ordered categorical outcomes” is mentioned. However, maximum mean discrepancy is reported. This method was not described in the appropriate section.

(b) An explanation as to why a modified Poisson approach was used instead of a logistic or log-binomial regression is needed. Similarly, the *Results* section shows an adjusted relative risk. However, this is a cross-sectional sample. The use of relative risk needs to be justified.

8. Data availability: Consider mentioning something regarding data availability.

##### Results

1. Tables are supposed to be stand-alone. Please add a footnote to Table 1 indicating your matching methodology. Consider adding the standardized mean difference to check the balance between cases and controls. Please tell the reader what you meant by the social distancing score scale. Please explain what is meant by patient activation. Please indicate whether the prescription is specific to hypertension.

2. Consider adding a supplementary table with the results from the follow-up period.

3. Table 2 results are not consistent with the proposed methods nor with the title of the table. Regression models result in exponentiated coefficients presented as odds ratios. In contrast, Table 2 shows MMD (or “MDD” for the social distancing score). Please present your MMD distributions in a separate table (or in the text) and introduce the appropriate methods in the previous section. Consider reporting IQR instead of 95% CI.

4. Please review the following numbers as they do not add up to 1005: “On average the hypertension sample thought that 7% of people who get COVID-19 would die as a result, and 63% would only experience mild symptoms.”

5. “On average the mean STAI was 1.90 units higher (95% CI 0.19-3.61, *P*=.03, Cohen *d*=0.13) for those with hypertension (40.75) than matched controls (38.85), with both groups higher than normal range, but below clinical levels.” The interpretation should be moved to the *Discussion* section. Please explain what you mean by “below clinical levels” as well as your reference scale.

6. Please clarify whether you adjusted for baseline characteristics in these analyses: “At follow-up, there was no longer a significant difference between the hypertension and control groups for influenza vaccination.”

##### Discussion and Conclusions

1. The discussion does not start by stating the study’s main findings (the influence of hypertension in the selected outcomes). Instead, it starts by comparing the overall sample with previous results in the same reference population.

2. The results are not discussed from multiple angles. For instance, the authors write, “Those with hypertension were more likely to take up the influenza vaccine during lockdown compared to healthy controls.” Could this be an effect of requiring care more often than healthy individuals? Patient activation is different from patient engagement.

3. The authors do not differentiate between willingness to get a vaccine and those who have already gotten a vaccine. Were there active vaccination campaigns between the two survey waves?

4. The authors mention several limitations of the study without detailing why they are limitations and how they were addressed. For instance, the authors write, “The sample was recruited via an online panel and social media, and has a low proportion of culturally and linguistically diverse participants.” What is the implication of this on the interpretation of your results? Did you do something to address such a shortcoming? Also, what are other implications?

5. Are people online more likely to be exposed to news generating anxiety or promoting vaccination? While this is just an example, most limitations lack this broader consideration.

6. Finally, conclusions are overextended and assume a causal effect: “Anxiety was above normal levels for all groups during the COVID-19 lockdown. This was higher in the hypertension group and appeared to translate to higher influenza vaccination intentions”; this is not consistent with the variable measured (intentions + uptake).

## Round 2 Review

### Specific Comments

The authors have addressed most if not all of the comments. I think the paper needs some proofreading, but that should not prevent its acceptance.
